# Social media and COVID-19 misinformation: how ignorant Facebook users are?

**DOI:** 10.1016/j.heliyon.2021.e07144

**Published:** 2021-05-26

**Authors:** Md. Sayeed Al-Zaman

**Affiliations:** a Department of Journalism and Media Studies, Jahangirnagar University, Savar, Dhaka, 1342, Bangladesh; b Department of Media and Technology Studies, University of Alberta, Edmonton, Alberta, T6G 2E6, Canada

**Keywords:** Social media, Ignorance, Misinformation, COVID-19, Facebook

## Abstract

The COVID-19 pandemic has claimed a lot of lives around the world, not only with the virus but also with misinformation. Many researchers have investigated COVID-19 misinformation, but none of them was related to social media users’ diverse responses to different types of COVID-19 misinformation, which could be a timely exploration. To bridge this gap in scholarly literature, the present study based on 11,716 comments from 876 Facebook posts on five COVID-19 misinformation seeks to answer two relevant research questions: (a) How ignorant social media users are about misinformation? (b) How do they react to different types of misinformation? Following a quantitative content analysis method, this study produces a few novel findings. The results show that most of the users trust misinformation (60.88%), and fewer can deny (16.15%) or doubt (13.30%) the claims based on proper reasons. The acceptance of religious misinformation (94.72%) surpassed other types of misinformation. Most of the users react happily (34.50%) to misinformation: the users who accept misinformation are mostly happy (55.02%) because it may satisfy their expectations, and the users who distrust misinformation are mostly angry (44.05%) presuming it may cause harm to people. The chi-square and phi coefficient values show strong positive and significant associations between the themes, levels of ignorance, and reactions to misinformation. Some strengths, limitations, and ethical concerns of this study have also been discussed.

## 1 Introduction

The COVID-19 pandemic is addressed as the largest scientific experiment in human history in many academic disciplines like criminology, economics, psychology, and biological sciences. It has also changed remarkably how we communicate with each other. Although digital media has been crawling into our daily lives for the last two decades, the pandemic brings a new occasion for it to flourish further. During the lockdown, in many countries around the world, consumption of digital media, such as social media, has increased by 65–75% (Casero-Ripolles, 2020; Keelery, 2020). On such a backdrop, what harm the internet with a huge flow of misinformation could cause can be easily predictable. In practice, online COVID-19 misinformation has been claiming lives worldwide (Coleman, 2020; Islam et al., 2020), urging scholars to investigate the relevant issues regarding COVID-19 misinformation. Although many studies have been conducted to date in the field of communication studies, psychology, and philosophy relating to the COVID-19 pandemic (Al-Zaman, 2021b; Casero-Ripolles, 2020; Cinelli et al., 2020; Laato et al., 2020; Naeem and Bhatti, 2020; Pennycook et al., 2020; Rovetta and Bhagavathula, 2020), none of them explored how social media users respond to different types of COVID-19 misinformation, and what their emotional expressions are when they interact with such misinformation. The present study attempted to bridge this knowledge gap a little. Based on two relevant and interrelated research questions and following a quantitative content analysis method, this study analyzed social media users’ comments on COVID-19 misinformation. The results yielded some novel insights.

The paper is divided into four main sections. In the background, the relevant concepts are discussed based on the previous literature. The methodology section presents the details of data collection and data analysis. The important results and pertinent interpretations are presented in the result section. The conclusion section sketches some limitations and strengths of the study.

## 2 Background

Information is meaningful data. In the study of the philosophy of information (PI), ethics occupies an important area, which asks various philosophical questions: Should information be true? Or, can it be false? Is it possible for us to always determine information's truth-value? How should we define and deal with information that tends to mislead a moral agent presenting false information? Is misinformation always false, or can it be true sometimes? Philosophers around the world are concerned about these inquiries and try to resolve such epistemic complexities on an acceptable ground, but little success has been achieved so far. For instance, it is still a contesting idea whether information has to be true or not. Floridi (2003, 2011) argued that information must have both meaning and truth-value: if one condition is violated, it cannot be information anymore. Dretske (1981) and Frické (1997) also agreed on this point that false information is a violation of the virtue of information. In contrast, Fetzer (2004) stated that information has *nothing* to do with truthfulness as we encounter a lot of information regularly and “we do not know whether it is true or false” (Fetzer, 2004). Fox (1983) also suggested that information needs not to be true, which differs from the views of Floridi, Dretske, and Frické. In terms of knowledge, while information that is true adds new knowledge, information that is not true adds no new knowledge (Floridi, 2010). What about misinformation? How should the relationship between misinformation and knowledge/ignorance be explained? Such questions are tried to be explained in the following sections, along with other important concepts based on the previous literature.

### 2.1 Misinformation & ignorance

The term *misinformation* has been used in scholarly literature widely after the 2016 US election (Quandt et al., 2019). While some theorists addressed misinformation as a *species* of information (Cooke, 2017; Fox, 1983), some argued misinformation is something else but information (Fallis, 2011, 2015; Floridi, 2011; Wardle, 2017). Thus, the scholars having diverged views are yet to produce a standard and acceptable definition of misinformation. As a result, most of the researchers have either used dictionary definitions or formulated their working definitions to conceptualize misinformation (Karlova and Fisher, 2013). Analyzing previous literature, we can identify three main attempts at conceptualizing misinformation.

The first concept addresses misinformation as a piece of information that has a lack of veracity but misleads people (Fallis, 2011; Floridi, 2011; Meel and Vishwakarma, 2020; Wardle, 2017). These scholars make a distinction between misinformation as unintentional and disinformation as intentional falsity. However, indeed, the intention of information cannot always be determined, just like it is nearly impossible to decipher the true value of every piece of information we encounter every day (Fetzer, 2004). The second group of scholars maintain a middle position addressing misinformation as either false or misleading or fabricated information, which is predominantly unintentional and *honest mistake* (Allcott and Gentzkow, 2017; Lazer et al., 2018): some used the relevant terms (i.e., rumor, misinformation, disinformation, and fake news) interchangeably as well (Al-Zaman et al., 2020; Duffy et al., 2020; Karppi et al., 2020; Tandoc et al., 2020) because, like truthfulness, it is sometimes difficult to assess the intention of information (Treen, Williams, and O'Neill, 2020). The third concept states that misinformation can be true and informative depending on the context (Cooke, 2017; Hollister, 2020; Karlova and Fisher, 2013; Karlova and Lee, 2011). For example, news articles sometimes contain information, especially the headlines in mediocre media outlets, which is not false but misleads readers, presenting true information: Such misinformation affects readers' memory, inferential reasoning, and behavioral intentions (Ecker et al., 2014). Also, in some definitions of misinformation, the idea of *intentionality* is missing, meaning misinformation can be both intentional and unintentional (Treen et al., 2020).

Assessing these three perspectives, we can address three main points of arguments in the concept of misinformation: it can be true or false; it can be intentional or unintentional; it always misleads people. In this paper emphasizing the third point, we used the following definition of misinformation: Misinformation is the “misleading information that is created and spread, regardless of whether there is intent to deceive,” (Treen et al., 2020, p. 4) and regardless of whether it is true or false.

Misinformation has a reciprocal relationship with knowledge and ignorance, and all are concerns of information ethics (Froehlich, 2017). All individuals are ignorant of some issues, and misinformation deepens it further. Put another way, while true and non-deceptive information is responsible for knowledge, misleading information is somewhat responsible for ignorance as it does not add new knowledge (Floridi, 2010). Although Fox (1983) argued that misinformation can be informative as well, he did not explain the propensity and applicability of such informativeness. To get a clearer picture of the interplay between misinformation and ignorance, we should demystify the concept of ignorance at first.

Ignorance is the opposite of knowledge. It can be defined from two perspectives: Standard View (SV) and New View (NV). SV states that ignorance is the absence of information (Smithson, 1989), whereas NV promotes that the information should be true. In another way, the lack of true belief is ignorance (Peels, 2017). Similarly, ignorance is believing false information or disbelieving true information (Dellantonio and Pastore, 2020). Though both SV and NV contradict at some points (e.g., when a person believes a true position without knowing it, it is ignorance in SV and not ignorance in NV), they show interdependence as well. Combining both views, in a broad sense, we can define ignorance as the lack of true information. In another word, a person believing misleading information is ignorant. Trusting misinformation reinforces a person's ignorance. Misinformation can be a reason for ignorance to some extent and can be an amplifier of ignorance to a greater extent. In this point, Froehlich (2017) argued that misinformation is a form and/or initiator of ignorance.

### 2.2 Ignorance, social media & COVID-19 misinformation

At the outset of the COVID-19 pandemic, the World Health Organization (WHO) declared that the pandemic is accompanied by an infodemic, i.e., an information pandemic. Infomdemic contains not only verified information but also distorted information, and filtering unreliable information becomes nearly impossible in the age of social media (Naeem and Bhatti, 2020). Meanwhile, thousands of people suffer from health hazards due to medication and health misinformation. For example, a piece of COVID-19 misinformation circulated globally claimed that highly concentrated alcohol could disinfect the body by killing the virus, and this misinformation killed at least 800 of its believers (M. S. Islam et al., 2020). Another online misinformation about drinking methanol as a cure of COVID-19 blinded 60 people and 5,876 had to be hospitalized (Coleman, 2020; M. S. Islam et al., 2020). The people who suffered from health hazards, firstly, were ignorant about COVID-19 cure and, secondly, misinformation intensified their ignorance, compelling them to engage in detrimental actions, i.e., taking wrong medications. Such instances around the world may clarify the connection between COVID-19 ignorance and COVID-19 misinformation. Social media is highly responsible for disseminating ignorance and misinformation worldwide not only during the pandemic but also for the last few years (Al-Biladi, 2016).

Many researchers studied the interplay between social media and COVID-19 misinformation from various aspects, such as users' information seeking and sharing behavior (Laato et al., 2020; Pennycook et al., 2020; Rovetta and Bhagavathula, 2020), cross-platform studies of misinformation (Cinelli et al., 2020), users' (mis)information consumption tendency (Casero-Ripolles, 2020). Regarding COVID-19 misinformation, Laato et al. (2020) showed that users share unverified information based on their trust in the source and due to information overload, although users' reliance on information sources was questioned in other studies (Marwick and Lewis, 2017). Also, this study did not focus on the knowledge level of the misinformation disseminators. In contrast, in a more relevant study, Pennycook et al. (2020) explored that *ignorance* is a crucial factor: many users share and believe misinformation because simply they fail to identify the truth-value of information. Another study shows that during the pandemic, people's information consumption rate increased from 60% to 92%, and their misinformation identification capacity increased by 12%, which suggests that the more information a person would consume, the more capable the person would be to identify misinformation (Casero-Ripolles, 2020). In *information-as-a-resource* ethics of the Resource-Product-Target (RTP) model (a model of information ethics), Floridi (2010) explained a similar idea that a moral agent requires quality information to make a more right decision: The more reliable information the agent gets, the better decisions the agent can make. That means if the ignorant persons who died from COVID-19 misinformation would have been still alive if they were supplied with more reliable information because it would prevent them from making poor decisions.

### 2.3 Users’ response to misinformation: can they identify misinformation?

Social media users evaluate the trustworthiness of information based on mainly its source, content, and who else shares or trusts the information (Flintham et al., 2018). However, studies also explored that sources can be less important than the information sharers and users’ self-motivation (Marwick and Lewis, 2017). Many researchers to date investigated how social media users respond to the misinformation they encounter in different platforms, whether they trust misinformation or deny it, and how. In the theory of rumor transmission, Buckner (1965) discussed the different standpoints of people as a recipient of a rumor. According to that, social media users can be of two main types: critical, uncritical, transmitter. The first group of users, based on a few traits, can critically evaluate a piece of information and can detect whether it is reliable or unreliable. On the contrary, the second group of users with a lack of critical ability cannot identify misinformation.

A study on the reactions of Bangladeshi social media users to religious misinformation explored that users mostly react emotionally than logically, and they mostly exhibit destructive behavior (Al-Zaman, 2021a). Tandoc et al. (2020) studied Singaporean social media users' response to misinformation. Following a mixed-method approach with a combination of survey and in-depth interview, the researchers identified most users ignore fake news when they encounter it on social media platforms. However, these users only offer corrections of fake news if it is either related to themselves or people with whom they have close interpersonal relationships (Tandoc et al., 2020, p. 12). This study did not discuss anything on users’ ignorance or knowledge level regarding misinformation.

Geeng et al. (2020) conducted similar research following an interview and participant observation method to identify how social media users interact with misinformation, why some users do not investigate the information they doubt, and how some of them investigate it. This study explored that users interact with misinformation in seven different ways: skip or ignore misinformation, accept misinformation content as face value, share or like misinformation, skeptical about misinformation, skeptical about misinformation's context, produce different perspectives, and misinterpret misinformation (Geeng et al., 2020, pp. 5–7). While this study attempts to provide some insights, it falls short in some points. First, the percentage of each response is absent in the study, which makes it difficult to understand what type of user response is more prevalent than others. Second, the responses could be more precise to avoid conceptual ambiguity, combining two or more categories into one. For example, *skepticism about misinformation* would be enough to define *skepticism about misinformation's context*, and it could be termed as *doubt*. Also, these responses do not provide a clearer picture about the valence of rationality: Do more users accept misinformation, or more of them reject it?

Ng and Loke (2020) overcame some limitations of the previous studies, providing some better perspectives regarding users' response to misinformation. They studied a Singapore-based Telegram group with more than 10,000 users to explore how they react to COVID-19 misinformation. The researchers categorized the users' response into four types: *affirm*, when users accept the misinformation; *denies*, when users refute the misinformation; *questions*, when users have doubts about the misinformation; *unrelated*, when users response is not relevant to the misinformation (Ng and Loke, 2020, p. 4). The study shows most of the users deny or question (45%) misinformation, while only a few users affirm (11%). This result advocates that social media users’ misinformation detection capacity outmatches their failure of misinformation detection.

In an experiment of misinformation detection capacity of human as an internet user and automated classifier, Kumar et al. (2016) found that human's misinformation detection capacity is much lower (66%) than automated classifier (86%), which somewhat contradicts the finding of Ng and Loke (2020). They also found that users tend to treat the information in shorter texts as misinformation than information in longer texts (Kumar et al., 2016, p. 599). Though this study has exceptional findings, its limit prevents the results to be much useful. For example, the researchers compared the detection capacity of humans and machines but did not explain more about the human's different levels of responsiveness and rationality. In another study, Verdizada (2017) explored that social media users usually feel more attracted by misleading information than true information, although they share unreliable information less than reliable information as it may hurt their reputation, Altay et al. (2020) found. In this case, however, users must identify the misinformation at first, and they only can decide afterward whether they should share it or not: this aspect is absent in the study of Altay et al. Further, like Kumar et al. (2016), the finding of Verdizada (2017) also differs from the finding of Ng and Loke (2020). Previous studies are limited in a few ways. First, they offer some conflicting findings. Second, their findings are not relevant to the COVID-19 pandemic situation and offer limited insights regarding pandemic misinformation, making it difficult to predict social media users' ignorance level and reactions to misinformation. Therefore, the present study seeks to answer the following question.

*RQ1:* How ignorant social media users are about misinformation?

Why can some users identify misinformation and others cannot? Buckner (1965) proposed three reasons why some people have the necessary critical ability to decipher misinformation: (a) If a person has the relevant knowledge regarding the topic of the misinformation; (b) if a person has familiarity with the similar situations in which misinformation usually arise; (c) if a person can evaluate the quality and reliability of the information source and overall information ecology (pp. 55–57). In the same paper, he also proposed five reasons why people trust misinformation: (a) If believing misinformation fulfills their needs; (b) due to the lack of relevant knowledge; (c) if an information vacuum is created and no other reliable information is available other than misinformation; (d) if nothing is known about a topic of public interest, but misinformation offers at least some explanation; (e) some people have inherent lacking of evidence evaluation capacity that make them victims of misinformation. From this conception, we can infer that believing or rejecting misinformation is dependent on two broad criteria: (a) a person's level of knowledge/ignorance (what s/he knows and does not know); (b) a person's relevant personal (what s/he wants and does not want) and situational (what types of information ecology s/he belong to) experiences.

### 2.4 Emotional reactions to misinformation

Although it is an important area of misinformation study, the previous researchers did not pay proper attention to social media users' emotional aspects and their interaction with misinformation. Emotional reaction has been considered an important area of psychological research, and social media researchers have recently adopted the idea of users' emotional investigations mostly by analyzing emojis (Al-Rawi, 2020; Al-Rawi et al., 2020). To frame the universal emotions, Ekman (1992), based on human facial expressions, developed his basic emotion theory consisting of six emotions: happiness, sadness, anger, surprise, fear, and disgust. More studies later showed that surprise and fear, and disgust and anger are similar in expressions (Jack et al., 2014). On the other hand, Russell (1980) extended the human emotions further, denoting 28 emotions in his circumplex model of affect. Previous studies borrowed these theories to analyze media texts. For example, Inkpen et al. (2000) analyzed newspaper headlines and text corpus based on Ekman's six emotions. In another study, Wen and Wan (2014), focusing on a lexicon-based approach, analyzed a microblog's text (i.e., Sina Weibo) based on a modified version of the basic emotion theory to understand the moods of the texts. Although many studies dealt with emotions in media texts, study on digital texts is still scarce to date. Moreover, the adoption of emotional theories to analyze social media users' reactions to misinformation is a relatively novel approach in communication studies. Therefore, this research attempts to answer the following question.

*RQ2:* How do social media users react to different types of misinformation?

## 3 Methodology

### 3.1 Data source & data collection

For this study, we used Facebook as the primary source of our data. We chose Facebook (94.46%) over other social media platforms, such as YouTube (3.31%) and Twitter (0.3%), because of its increasing popularity in Bangladesh. We analyzed popular misinformation from three fact-checking websites: BD Factcheck (www.bdfactcheck.com), Boom Live (www.boombd.com), and Jachai (www.jachai.org). All are non-profit Bangla fact-checking websites, run and contributed by Bangladeshi media researchers and professionals. Their rigorous misinformation debunking methods make their data useful for research purposes (Al-Zaman, 2021c; Al-Zaman et al., 2020). Also, collecting data from fact-checking websites for research purposes has become popular nowadays (Avaaz, 2019; Brennen et al., 2020; Kanozia et al., 2021). Note that in December 2020, Facebook has declared to remove all COVID-19 misinformation that may lead to health catastrophe (Issac, 2020). For that reason, Facebook has removed many prevalent pieces of misinformation along with their metadata. However, we found that some misinformation was still available on Facebook but flagged as misleading information. Therefore, before selecting any misinformation, we checked the availability of that misinformation on Facebook through searching. Following the five most popular themes of misinformation (i.e., health, religion, politics, crime, and entertainment) (Al-Zaman, 2021b), we finally selected five popular and available misinformation listed on the fact-checking website: one misinformation from each theme. A few previous studies also identified popular misinformation themes. For example, Brennen et al. (2020) identified nine themes of COVID-19 misinformation analyzing 225 pieces of misinformation, and Sutaria (2020) identified two major themes of COVID-19 misinformation based on 178 pieces of Indian misinformation. However, none of these studies were relevant to the context of Bangladesh.

Using predetermined keywords and phrases consistent with the misinformation claims (see Table 1), we searched on Facebook for the posts containing misinformation. For searching purposes, we used CrowdTangle, a public insights tool owned and operated by Facebook. We searched pages and public groups for the available posts written in Bangla. The timeframe of our search was from 1 February 2020 to 1 February 2021, 12 months in total. During this period, the selected five misinformation produced 876 posts and 569,948 interactions (Mean (M) = 650.63) (Table 1). These data were accessed on 12 February 2021.

**Table 1:** Metadata of the selected misinformation.

Themes	Claims^a^	Search keywords and phrases^b^	Posts	Interactions	Total comments	Mean, Standard Deviation	Selected comments
Health	Ethanol will prevent corona	*Ethanol a dur hobe corona*	18	194,170	2,454	136.33, 581.31	291 (11.86%)
Religion	*Oju* can prevent COVID-19	*Oju holo corona virus er sorbottom oshud*	99	155,355	1,494	15.09, 28.05	303 (20.28%)
Politics	Thirty-five countries will sue China	*Chiner biruddhe mamla korbe 35 ti desh*	572	105,104	5,703	9.97, 34.96	258 (4.52%)
Crime	Twenty million people are missing in China	*China te 20 million manush nikhoj*	98	69,224	751	7.66, 27.71	302 (40.21%)
Entertainment	Corona positive vs. negative football match	*corona positive vs. corona negative football*	89	46,095	1,314	14.76, 108.70	252 (19.18%)
Total			876	569,948	11,716	13.74, 126.32	1406 (12%)

a The claims were collected from Bangladeshi fact-checking websites.

b The search terms and phrases were in Bangla since most of the Bangladeshi social media users use the Bangla fonts on Facebook. These were consistent with the claims.

In the data collection phase, we collected publicly available comments both manually and using Comment Exporter (http://commentexporter.com/). The comments were separated into different sheets in the Excel file according to their thematic categories. From these sheets, following a simple random sampling technique, we selected a sample of 1,850 comments (15.89% of the total comments) from a total of 11,716 comments. To elaborate more, we selected random numbers of comments from each theme and analyzed them separately for RQ2. But for RQ1, we calculated them combinedly. In this regard, as it was a simple random sampling, we did not adhere to any specific percentage of comments from each theme. Therefore, the percentages of comments varied across themes, which could have been a limitation of the sampling if we had not calculated only the *intra*-themes’ (row) percentages instead of *inter*-themes’ (column) percentage. We used simple random sampling because this sampling technique allows researchers to “generalize information legitimately from a few [comments]” (Neuman, 2011, p. 49). We further excluded 444 comments from the count, which included comments that were either irrelevant (e.g., advertisement, link, sticker, mention) or missing. We also excluded the duplicate comments using the Duplicate option in Excel. Our final sample for this study was 1,406 comments. The unit of analysis was each comment. A comment can express more than one meaning at a time. In such cases, we decided to code one prevalent meaning for each comment, relying on the coders' intuition. We used Microsoft Excel 2019 to compile the comments and other metadata and to code the data. Note that in this study, we analyzed only users’ public comments, excluding other reactions, such as emojis.

### 3.2 Data analysis & preparing codebook

In this quantitative content analysis, we built our codebook based on previous research before collecting the data. Afterward, we applied a deductive or fixed coding approach. To answer the first question, we derived four codes from Ng and Loke (2020) and Geeng et al. (2020) with little modifications: accept, deny, doubt, and other. With the code *accept*, we indicated the tendency of believing the claims of misinformation; with *deny*, we indicated users' refutation of the misinformation claims based on proper, improper, or no reasoning; with *doubt*, we indicated users’ dilemma regarding the claim whether they should believe it or reject it; the comments that did not fit into the previous three definitions were coded as *other*.

For the second question, we derived six emotional reactions from the basic emotion theory of Ekman (1992): happy, sad, angry, fear, disgust, and surprise, adding a seventh code *none* to enlist the emotions that expressed something else than the six main emotions (Inkpen et al., 2000; Wen and Wan, 2014). Worth mentioning that many theorists addressed the circumplex theory of emotion (Posner et al., 2005; Russell, 1980) as more effective than the basic emotion theory to understand both the emotional valence (positive and negative) and the emotional arousal (high and low) (Gu et al., 2019). We presumed that a synthesis of both theories would benefit the understanding of users' reactions more clearly. Therefore, we redefined the six basic emotions as follows with a few more emotions deriving from the circumplex model: happy (amused, proud, confident, satisfied, contented, and comforted), sad (miserable, guilt, and depressed), angry (disturbed, restlessness, and affliction), fear (terror and agitated), disgust (embarrassed, contempt, and confused), and surprise (interest, shock, and aroused) (Sarraipa et al., 2016). Of the six major reactions, all the five except happy indicate negative valence: only happy indicates positive valence. Regarding the coding, the author of this paper along with one trained coder coded 7.11% (100 comments) of the sample. The coding issues were resolved based on mutual consent. For interrater reliability, Cohen's Kappa value for the levels of ignorance was found κ_1_ = 0.857 and for the reactions to misinformation was found κ_2_ = 0.871. Both values indicate almost perfect agreements (Gisev et al., 2013). The statistical analysis for this study was conducted using IBM SPSS Statistics version 25.

## 4 Result & discussion

This study sought to answer two research questions by analyzing Facebook users' comments on five different types of COVID-19 misinformation. The first question aimed at understanding the levels of users’ knowledge regarding COVID-19 issues, and the second question wanted to explore how users react to different misinformation based on their levels of knowledge.

The result shows that most of the users (*n =* 856; 60.88%) tend to accept the claims of misinformation, with a very lower number of users (*n =* 227; 16.15%) who can refute misinformation either based on knowledge or personal experiences (Table 2). On the other hand, only a small number of users (*n =* 187; 13.30%) were doubtful about the claims of misinformation. These results challenge the findings of Ng and Loke (2020), and partially consistent with the findings of Kumar et al. (2016) and Verdizada (2017). Most of the users accepting misinformation indicate that most of them have insufficient knowledge and relevant experiences to determine what is right and what is wrong (Buckner, 1965) and, more precisely, they are ignorant of the particular misinformation issues. It may have two reasons. First, these users were not supplied with more reliable information about the misinformation issues before they confronted them, so they failed to make the right decision, i.e., to deny misinformation (Floridi, 2010). Second, they might have a lack of information literacy, which prevents them from identifying the real nature of the misinformation they encounter on Facebook. Information literacy includes cross-checking and analyzing the source. In reality, most of the social media users in Bangladesh and India indeed lack digital information literacy (R. Islam, 2018), and it is one of the main reasons for the prevalence of misinformation in these two countries (Gogon, 2021; Raj and Goswami, 2020). Most of the users who denied or doubted misinformation proposed no explanation behind their rejection or doubtfulness, which infers that they either did not know why the information is *misinformation* or they were reluctant to offer corrections like Tandoc et al. (2020) found in their study. A few of them argued against misinformation based on reasons: such deniers mostly stated, for example, that in what ways ethanol can be dangerous for human health and what harms it can cause. Like most of the deniers, most believers of misinformation also offered a few arguments or reasons behind their choice. Believers of information, for instance, argued that the government should promote the native medication (i.e., the idea of ethanol) of coronavirus instead of promoting foreign endeavors (vaccine nationalism; Khan, 2021), that *oju* (*wudu* in Arabic, which refers to the cleansing of body parts) alone can really prevent coronavirus (willful ignorance; Lynch, 2016), and that coronavirus is the mechanism of China (xenophobia; Mamun and Griffiths, 2020). These are the instances of emotional response. Considering these responses, it is partly evident that most users’ response to misinformation is not guided by proper reasons (Al-Zaman, 2021a).

**Table 2:** Frequencies and percentages of different levels of ignorance and reactions to misinformation.

Levels of ignorance	Frequency (*n*)	Percentage (%)	Reactions to misinformation	Frequency (*n*)	Percentage (%)
Accept	856	60.88%	Happy	485	34.50%
Deny	227	16.15%	Sad	59	4.20%
Doubt	187	13.30%	Angry	201	14.30%
Other	136	9.67%	Disgust	205	14.58%
			Fear	81	5.76%
			Surprise	118	8.39%
			None	257	18.28%

The thematic misinformation analysis shows that religious misinformation (94.72%) has the highest acceptance to the users compared to the percentages of other misinformation, followed by entertainment (72.22%) and crime misinformation (60.26%) (Table 3). Interestingly, all except a very few users cannot resist religious information: only 2.31% denied and 0.66% doubted it. It suggests that religious misinformation in Bangladesh is like a *magic bullet* and Facebook users can hardly prevent it. Previous studies also showed the prevalence and trustworthiness of religious misinformation in Bangladesh and its aftermath in society (Al-Zaman et al., 2020). On the other hand, users are more competent in identifying and/or denying political misinformation (35.27%): users are more doubtful (24.81%) about political misinformation as well compared to others. It indicates that Bangladeshi Facebook users are politically conscious, as well as knowledgeable. This result can be explained from two perspectives: (a) Bangladesh is a hybrid regime with no strong opposition, and people likely have less trust in the government, which makes them more politically conscious and critical: online political memes and satires can be counted as evidence; (b) politicians are responsible for more political misinformation in Bangladesh, and due to the political distrust and critical position, users can easily demystify misinformation. In health misinformation, believers (45.70%) surpassed the deniers (16.84%) or doubters (18.90%). Such higher trust in health misinformation in Bangladesh could be detrimental to public health. A chi-square test (χ^2^ = 341.688) between the themes of misinformation and the levels of ignorance shows that both variables are correlated, and phi-coefficient (ϕ = 0.493) shows a strong positive and significant correlation between the variables with a *p*-value 0.000 (*p* < 0.05).

**Table 3:** Levels of ignorance based on different themes of misinformation.

Themes of misinformation	Levels of ignorance (*n*, %)			
	Accept	Deny	Doubt	Other
Health	133 (45.70%)	49 (16.84%)	55 (18.90%)	54 (18.56%)
Religion	287 (94.72%)	7 (2.31%)	2 (0.66%)	7 (2.31%)
Politics	72 (27.91%)	91 (35.27%)	64 (24.81%)	31 (12.02%)
Crime	182 (60.26%)	47 (15.56%)	51 (16.89%)	22 (7.28%)
Entertainment	182 (72.22%)	33 (13.10%)	15 (5.95%)	22 (8.73%)

Regarding the users' emotional reactions to misinformation, most of them reacted with happiness (*n =* 485; 34.50%) (Table 2). Of the other five emotions, users expressed disgust (*n =* 205; 14.58%) more often, followed by anger (*n =* 201; 14.30%). This result suggests that users’ emotional valence is more positive than negative when they encounter misinformation. It further suggests that the prevalence of destructive behavior among Facebook users is comparatively lesser than the previous studies presumed (Al-Zaman, 2021a). Interestingly, we found 257 comments (18.28%), which is the second-highest reaction on the list, that expressed reactions beyond the six basic emotions. These comments contained mainly the following reactions: speculation, ridicule, suggestive, justification, non-reactive; so, further misinformation research should explore these reactions.

Users who trusted misinformation tended to react happy (*n =* 471; 55.02%), followed by none (*n =* 95; 11.10%) and angry (9.81%): they expressed fear (*n =* 34; 3.97%) the least (Table 4). This result primarily shows that misinformation unknowingly can satisfy the users, justifying that in a situation of an information vacuum, misinformation feeds the appetite of the public (Difonzo and Bordia, 2006). Unlike the believers, however, most of the users who denied the claims of misinformation expressed anger (*n =* 100; 44.05%), followed by disgust (*n =* 63; 27.75%): they expressed the least amount of happiness and sadness (both *n =* 2; 0.88%). It suggests that users do not like misinformation when they can identify it, and it arouses mostly anger and to some extent discontent in them. On the other hand, doubtful users mostly expressed disgust (*n =* 60; 32.09%) and surprise (*n =* 47; 25.13%). Here in both cases, disgust denotes confusion as well, which we defined previously (see Methodology). That means misinformation produces not only anger among the deniers but also confusion among both deniers and doubters. A chi-square test (χ^2^ = 805.324) between the levels of ignorance and the reactions to misinformation shows that both variables are correlated, and phi-coefficient (ϕ = 0.757) shows a very strong positive and significant correlation between the variables with a *p*-value 0.000 (*p* < 0.05).

**Table 4:** Levels of ignorance according to different reactions.

Levels of ignorance	Reactions to misinformation (*n*, %)						
	Happy	Sad	Angry	Disgust	Fear	Surprise	None
Accept	471 (55.02%)	50 (5.84%)	84 (9.81%)	71 (8.29%)	34 (3.97%)	51 (5.96%)	95 (11.10%)
Deny	2 (0.88%)	2 (0.88%)	100 (44.05%)	63 (27.75%)	20 (8.81%)	6 (2.64%)	34 (14.98%)
Doubt	2 (1.07%)	4 (2.14%)	10 (5.35%)	60 (32.09%)	19 (10.16%)	47 (25.13%)	45 (24.06%)
Other	10 (7.35%)	3 (2.21%)	7 (5.15%)	11 (8.09%)	8 (5.88%)	14 (10.29%)	83 (61.03%)

In terms of emotional reactions, health misinformation (31.27%) received mostly happy reactions, followed by entertainment misinformation (28.17%), although religious misinformation (92.74%) surpassed others in percentage (Table 5). Happy users were mostly concerned with the positive misinformation claim, i.e., the invention of the COVID-19 vaccine by a Bangladeshi scientist. However, some users were: *angry* because other users were not supporting the vaccine initiative, or news outlets were spreading vaccine misinformation; *disgusted* and *confused* because they were unable to correctly identify the true value of the information, or some were believing the misinformation; *fearful* because such health misinformation could be a threat to many people. Crime misinformation received relatively more reactions of sad (9.27%), fear (9.27%), and surprise (13.91%) than other themes. However, in political (24.81%) and crime (23.84%) misinformation, most of the reactions were documented in *none* category, which means in many occasions for political and crime misinformation, users’ reactions do not correspond to the guiding codes. Also, many users (24.03%) reacted angrily against political misinformation because most of them could identify the misinformation, and the least number of users (1.98%) reacted angrily against religious misinformation because most users were perhaps willfully ignorant. A chi-square test (χ^2^ = 693.739) between the themes of misinformation and the reactions to misinformation shows that both variables are correlated, and phi-coefficient (ϕ = 0.702) shows a very strong positive and significant correlation between the variables with a *p*-value 0.000 (*p* < 0.05).

**Table 5:** Reactions to misinformation based on different themes of misinformation.

Themes of misinformation	Reactions to misinformation (*n*, %)						
	Happy	Sad	Angry	Disgust	Fear	Surprise	None
Health	91 (31.27%)	7 (2.41%)	44 (15.12%)	38 (13.06%)	26 (8.93%)	18 (6.19%)	67 (23.02%)
Religion	281 (92.74%)	1 (0.33%)	6 (1.98%)	2 (0.66%)	00 (00%)	1 (0.33%)	12 (3.96%)
Politics	25 (9.69%)	14 (5.43%)	62 (24.03%)	41 (15.89%)	20 (7.75%)	32 (12.40%)	64 (24.81%)
Crime	17 (5.63%)	28 (9.27%)	48 (15.89%)	67 (22.19%)	28 (9.27%)	42 (13.91%)	72 (23.84%)
Entertainment	71 (28.17%)	9 (3.57%)	41 (16.27%)	57 (22.62%)	7 (2.78%)	25 (9.92%)	42 (16.67%)

Table 6 shows that the users who believed health misinformation was mostly happy (67.67%) and least fearful (1.50%) because of their optimistic view toward the misinformation. Similarly, those who denied health misinformation were mostly angry (40.82%) and least happy (0%) because of their discontent regarding the probable consequences of the misinformation. The believers of religious misinformation expressed intense happiness (97.91%) with minimal anger (0.35%). On the other hand, the deniers of religious misinformation (57.14%) were mostly angry like the deniers of health (40.82%) misinformation, because many users were against the vaccine invention by local scientists and unable to understand the importance of religious functions like *oju*, respectively. The deniers of crime (46.81%) and entertainment misinformation (54.55%) were mostly disgusted. Like health misinformation, users who believed political (30.56%) and entertainment misinformation (33.52%) were mostly happy. Interestingly, users who reacted *happy* to crime misinformation expressed communalism, xenophobia, ethnic hatred, and nationalism.

**Table 6:** Thematic cross-tabulations between levels of ignorance and reactions to misinformation.

Themes & levels of ignorance	Reactions to misinformation (%)						
	Happy	Sad	Angry	Disgust	Fear	Surprise	None
*Health*							
Accept	67.67%	4.51%	12.03%	3.01%	1.50%	4.51%	6.77%
Deny	0.00%	2.04%	40.82%	10.20%	34.69%	4.08%	8.16%
Doubt	1.82%	0.00%	7.27%	45.45%	9.09%	10.91%	25.45%
Other	0.00%	0.00%	7.41%	7.41%	3.70%	7.41%	74.07%
*Religion*							
Accept	97.91%	0.00%	0.35%	0.00%	0.00%	0.00%	1.74%
Deny	0.00%	0.00%	57.14%	14.29%	0.00%	0.00%	28.57%
Doubt	0.00%	0.00%	0.00%	0.00%	0.00%	50.00%	50.00%
Other	0.00%	14.29%	14.29%	14.29%	0.00%	0.00%	57.14%
*Politics*							
Accept	30.56%	11.11%	11.11%	1.39%	11.11%	12.50%	22.22%
Deny	1.10%	1.10%	56.04%	18.68%	2.20%	3.30%	17.58%
Doubt	1.56%	6.25%	4.69%	32.81%	7.81%	21.88%	25.00%
Other	3.23%	3.23%	0.00%	6.45%	16.13%	19.35%	51.61%
*Crime*							
Accept	9.34%	14.84%	18.13%	17.58%	9.34%	10.99%	19.78%
Deny	0.00%	0.00%	27.66%	46.81%	2.13%	2.13%	21.28%
Doubt	0.00%	0.00%	1.96%	19.61%	17.65%	39.22%	21.57%
Other	0.00%	4.55%	4.55%	13.64%	4.55%	4.55%	68.18%
*Entertainment*							
Accept	33.52%	4.95%	14.29%	18.68%	3.85%	8.79%	15.93%
Deny	3.03%	0.00%	36.36%	54.55%	0.00%	0.00%	6.06%
Doubt	0.00%	0.00%	13.33%	26.67%	0.00%	40.00%	20.00%
Other	40.91%	0.00%	4.55%	4.55%	0.00%	13.64%	36.36%

## 5 Conclusion

To conclude, this research is limited in a few ways. First, it only considered the public comments to understand users' ignorance and reactions to general and specific types of misinformation, excluding other expressive indicators like emojis. Studies on major Facebook reaction buttons demonstrated the communicative and expressive values of such non-verbal digital elements (Al-Rawi, 2020). Second, this study is limited in the context of Bangladesh like the previous studies, such as Tandoc et al. (2020) and Ng and Loke (2020) as both focused on Singapore. Also, Bangladesh's online community should be different from other countries in a few ways. Therefore, the results may not be generalized to other countries. Third, analysis with a larger dataset or more cases of misinformation might produce different results. Also, unlike the present study, most of the previous studies on COVID-19 misinformation dealt with misinformation claims and contents (Al-Zaman, 2021b; Brennen et al., 2020; Sutaria, 2020). Finally, this study is unable to collect and analyze the sociodemographic information of the users because (a) Facebook's account privacy often do not let such information publicly available, (b) collecting such data is time-consuming, and (c) accessing such data either manually or via API (Application Programming Interface) can potentially violate the users' right to privacy. Also, an analysis of sociodemographic data was not important for and aim of this study as well.

However, beyond these limitations, this study has some theoretical and practical values as well. First, Bangladesh and the Bangla language have been largely ignored in the global social science and humanities research streams. Although a few studies previously dealt with different aspects of Bangladeshi media and the COVID-19 situation, none of them considered the digital contents produced in Bangla, which has been a limitation in the academic scholarship: the present study bridged it a little. Second, previous studies failed to explain the public reactions to different thematic COVID-19 misinformation in social media. Although a few researchers attempted to provide some insights (Ng and Loke, 2020), their studies were limited in many ways. In contrast, this study explained the knowledge levels of social media users regarding COVID-19 issues and why they react differently to different thematic misinformation. Finally, the results would help to understand the behavioral patterns of Bangladeshi online communities regarding misleading information.

The data collected and used in this study demand some ethical clarifications as well. Social media data (e.g., Twitter and Facebook) have become popular in recent times, leaving room for ethical concerns from the aspects of data collection and data protection. For example, Franzke et al. (2020) argued that *informed consent* is needed regarding “automated scraping for semi-public data and the use of API processes for accessing private data” (p. 10). Also, the comments analyzed in the present study were anonymized for users' privacy protection. From this, it seems that the collection of public data is exempted from the ethical prohibition. Mancosu and Vegetti (2020) detailed the ethical issue regarding Facebook data into two stages: data preparation period and data reporting period. The first stage includes collecting, storing, cleaning, and analyzing the data. From an ethical standpoint, it is prohibited to collect, store, and analyze private data, such as private Facebook profiles. The second stage includes the publishing of data in either analyzed or raw format. In such cases, researchers are often advised not to reveal the names of the users. From both perspectives, our research data met the ethical standards as we collected publicly available data for the research purposes and removed users’ names from the final dataset.

## Declarations

### Author contribution statement

Md. Sayeed Al-Zaman: Conceived and designed the experiments; Performed the experiments; Analyzed and interpreted the data; Contributed reagents, materials, analysis tools or data; Wrote the paper.

### Funding statement

This research did not receive any specific grant from funding agencies in the public, commercial, or not-for-profit sectors.

### Data availability statement

The data that has been used is confidential.

### Declaration of interests statement

The authors declare no conflict of interest.

### Additional information

No additional information is available for this paper.
